# An online evidence based medicine exercise prompts reflection in third year medical students

**DOI:** 10.1186/1472-6920-14-164

**Published:** 2014-08-09

**Authors:** Linda Orkin Lewin, Nancy J Robert, John Raczek, Carol Carraccio, Patricia J Hicks

**Affiliations:** 1 Department of Pediatrics, University of Maryland School of Medicine, 22 South Greene Street, 21201 Baltimore, MD, USA; 2 American Nurses Association, 8515 Georgia Avenue, 20910 Silver Spring, MD, USA; 3 Instructional Technology Group, University Of Maryland School Of Medicine, 655 West Baltimore Street, 21201 Baltimore, MD, USA; 4 American Board of Pediatrics, 111 Silver Cedar Court, 27514 Chapel Hill, NC, USA; 5 The Children’s Hospital of Philadelphia and the Perelman School of Medicine at the University Of Pennsylvania, 34th & 19104 Philadelphia, PA, USA

**Keywords:** Clinical decision making, Evidence based medicine, Medical student education, Online education, Reflection

## Abstract

**Background:**

Reflective practice is a desirable trait in physicians, yet there is little information about how it is taught to or learned by medical students. The purpose of this study was to determine whether an online Evidence Based Medicine (EBM) exercise with a face-to-face debriefing session would prompt third year medical students to reflect on their current skills and lead them to further reflection on clinical decision making in the future.

**Methods:**

All third year medical students at the University Of Maryland School Of Medicine who completed their pediatrics clerkship between 7/1/09 and 2/11/11 were required to complete the EBM exercise. Following completion each student received a personal report (Learning Profile) of their responses and attended a one hour large group debriefing session. Student responses to a survey following the debriefing sessions were analyzed using a post-test survey design with a single experimental cohort.

**Results:**

Ninety-five percent of students completing the debriefing survey indicated that the debriefing session helped them better understand their learning profiles; 68% stated that their profiles allowed them to evaluate themselves and their decisions. Sixty-three percent noted that participating in the exercise and the debrief would lead them to either learn more about EBM and use EBM more in the future or reflect more on their own decision making.

**Conclusions:**

The EBM exercise was a successful way to introduce the concept of reflective practice to third year medical students, and the graphic Learning Profiles were effective instigators of discussion and reflection.

## Background

Mindful practice in medicine is described as the process of becoming aware of one’s own thoughts, listening attentively to others, and remaining flexible in the approach to one’s work, allowing a physician to act with principles and compassion [1]. Awareness of one’s own thoughts requires reflection, a skill that has become the focus of attention of many medical educators in recent years. While reflection is often listed as a desirable ability in physicians [2,3] there is no single theory that explains how one develops this skill or what educational programs should do to promote it.

There are several approaches to fostering reflection in medical students that have been described; many of those revolve around some sort of writing assignment, with or without face-to-face or written feedback. Some curricula require a written response to a prompt, others ask for students to analyze real life experiences that they found challenging [4-8]. Several programs use student responses to art, literature, and even movie clips as prompts to reflect [9-12]. Each program strives to produce the conditions under which students will engage in the sometimes uncomfortable act of reflection in order to become more mindful in their practice going forward.

Despite this variety of curricular approaches, strong evidence supporting the optimal educational program to teach reflection is lacking. There are some hints in the literature, however, of approaches that offer the promise of fostering reflective practice in learners across the medical education continuum from medical student through practicing physician. In their review of the topic, Mann et al. suggest two pre-requisites to stimulate reflection: 1) an “authentic context” and 2) a complex clinical problem or anticipating an upcoming challenging situation [3]. The impetus for learning and reflection is based on the learner’s ability to “make sense of the world”, a process influenced by doubt, uncertainty, or perceived difficulty.

Similarly, Thompson et al. studied medical students’ cognitive and emotional processes during episodes of reflection and found that reflection was often triggered by some dissonance between their own values and those demonstrated by others in their environment [13]. The authors suggest that educators can promote reflection by creating educational activities that either create such conflicts or that bring them to light. Once the imperative to reflect is created, educators can guide learners through a reflective process in order to reconcile the two sets of values.

In fact, the presence of facilitators to help learners reflect is considered key by many authors [5,6,9,14,15]. Baernstein et al. compared the quantity and quality of medical student reflection on professionalism when they: 1) completed written critical incident reports, 2) combined completion of those reports with a discussion of the incident with a faculty member, and 3) engaged in a discussion of the incident without completing a written report. They found that the one-on-one discussion led to more reflection, with or without a written incident report, further supporting the importance of feedback and guidance on reflection in students [4].

Another study also found that that the feedback that medical students received on their written reflections were seen as a critical element by the students themselves [7]. Many medical schools have turned to portfolio systems to create substrate for student reflection, with mandated periodic review with an advisor allowing students to develop and practice their reflective skills over time [16]. The presence of such a supportive environment appears to be a critical element in actively promoting reflection [17].

Thus, reflection seems to be best achieved in medical students when a situation can be found or created that feels authentic to the learner, where some conflict between internal and external values or a critical question has been raised, and when there is a culture of reflection, a structured process for reflecting, and strong mentorship to support the reflective process.

The Evidence Based Medicine (EBM) exercise at the University Of Maryland School Of Medicine is completed by third year students as part of their third year clerkship in pediatrics. The exercise consists of an online survey that generates an individual report (Learning Profile) of responses for each participant. The report consists of graphs and charts that depict students’ self-efficacy beliefs regarding EBM calculations in relation to their actual skill in those calculations, as well as the factors that contributed to their problem solving in a hypothetical pediatric patient scenario. The profile serves as the starting point for a group discussion about the differences in students’ beliefs about their own abilities and decision making and their true performance. This one hour debriefing session is facilitated by the pediatric clerkship director and allows reflection in a safe and supportive setting.

The purpose of this study was to determine whether the students themselves felt that the debriefing session enhanced their understanding of and reflection on their Learning Profile, and whether engaging in the EBM exercise and debriefing would lead them to further reflection on clinical decision making in the future.

## Methods

### Description of exercise

The original EBM exercise and Learning Profile design were based on studies of the barriers to physician adoption of EBM practices and prior research suggesting that individuals’ attitudes and beliefs affect the ways that they evaluate information and adopt new skills [18-20]. Robert’s pilot study demonstrated that participants had gaps between their self-efficacy and actual performance around EBM tasks and made clinical decisions using a wide variety of cognitive processes that were influenced by more than just scientific evidence [21].

The survey and reporting process were modified for use by medical students and pediatric residents [22], as well as adapted as an electronic curriculum and placed on the website of the University Of Maryland School Of Medicine. The survey collection tool and data warehouse were set up within the University Of Maryland School Of Medicine’s existing student intranet infrastructure (Microsoft ASP.NET 3.5, IIS 7, and SQL Server 2008). The individualized student reports were created and delivered under the same infrastructure. The bar and pie charts were incorporated into each report dynamically using third-party charting software (Dundas Chart for NET).

The survey is currently available to students through the password protected website, and also publicly available at http://www.docdp.org/ebm/ebm.aspx?id=1&group=1&mode=delivery. It includes two sections; in the first, “EBM Self-Efficacy and Skill”, participants were asked how confident they are that they can perform 12 distinct EBM tasks and then were given 12 multiple choice questions, each related to performing one of those tasks, including some statistical calculations and some interpretation of results. In the second section, the “Pediatric Justification Case”, participants read the case of a 7 year old boy with signs and symptoms of Attention Deficit Hyperactivity Disorder and were given data based on a variety of sources (meta-analyses, lay literature, opinions of experts, and others) and asked to decide whether to treat the patient with methylphenidate, with behavior therapy, with both, or with neither. They then answered a series of questions that helped define how they reached their treatment decisions.

### Learning profile contents

**Figure 1:**
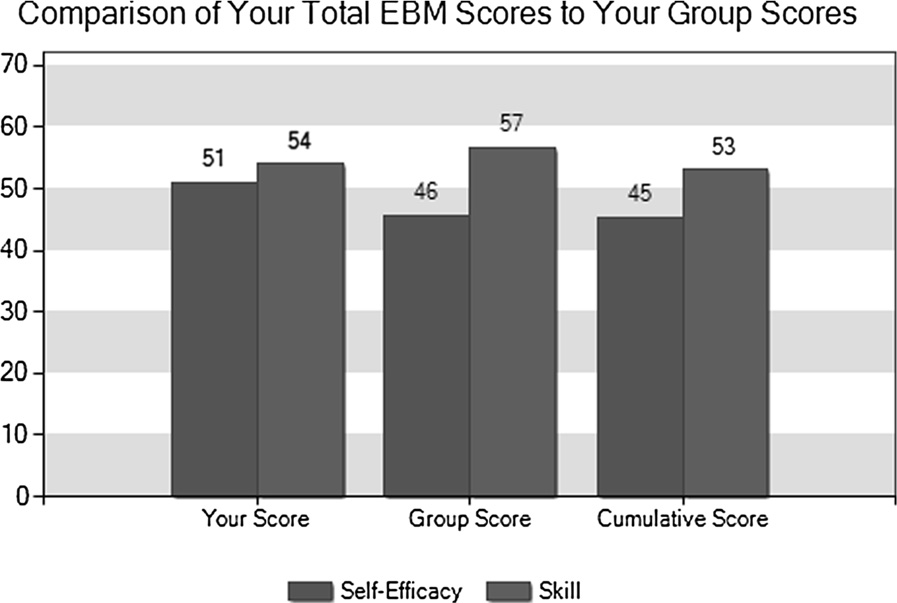
Learning profile representation of One Student’s overall self-efficacy vs. EBM skill.

**Figure 2:**
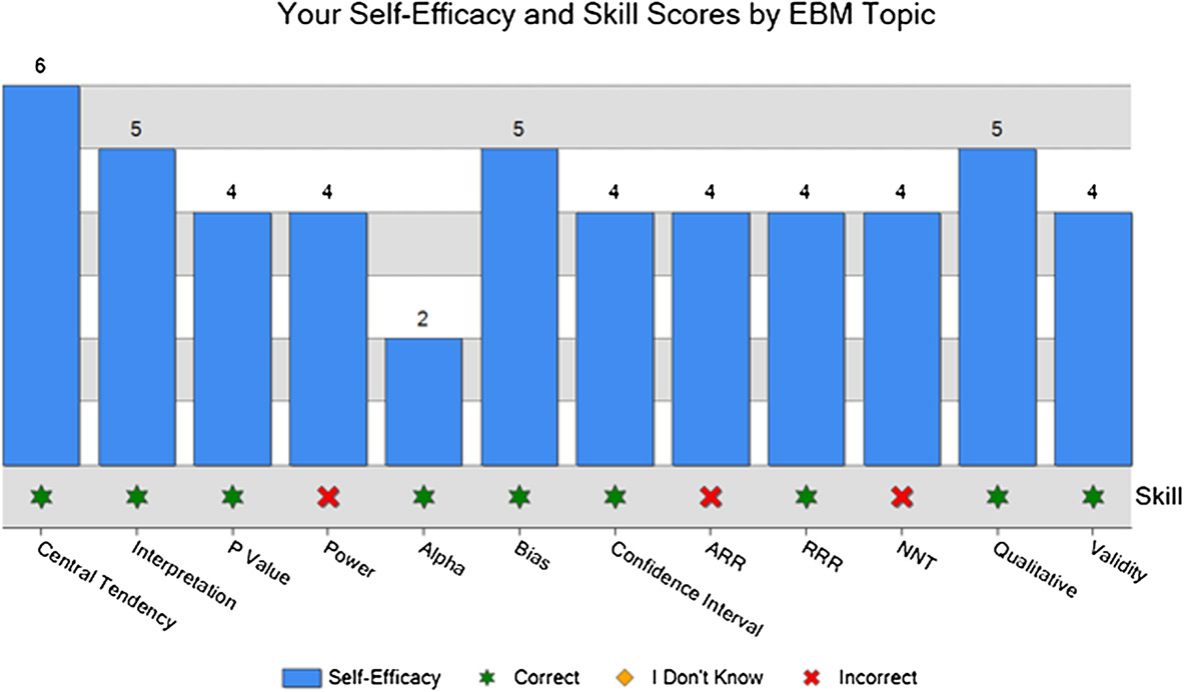
Learning profile representation of One Studen t’s self-efficacy vs. Skill, individual EBM tasks.

**Figure 3:**
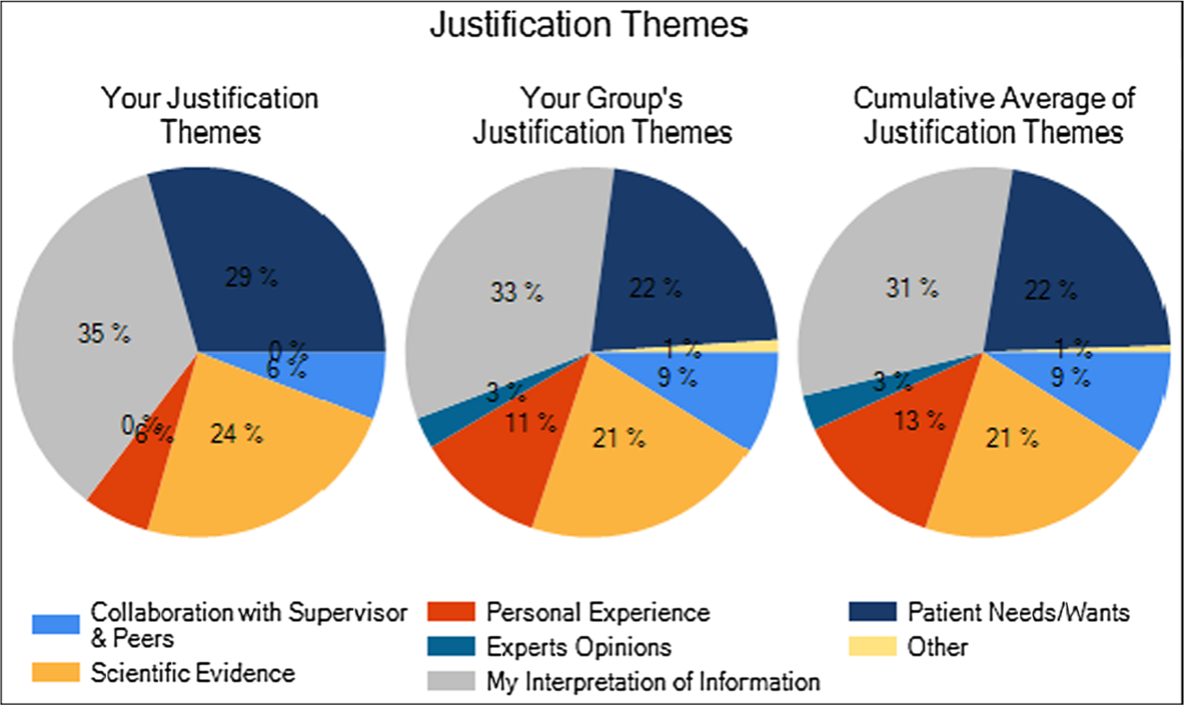
Learning profile representation of One Student’s Pie chart representation of case decision making.

The Personal Learning Profiles provided to the students contain graphic representations of their responses to the survey. Figures 1 and 2 depict the way that data on EBM self-efficacy and skill are presented. In Figure 1 the set of bars on the left represent the individual student’s results; the bar on the left represents the student’s self-efficacy score, or how confident he was that he could complete 12 specific EBM tasks. The bar to the right is that student’s actual performance of those 12 tasks. Students own scores are shown next to those of their entire clerkship group and those of all University of Maryland clerkship participants who have completed the survey since July, 2008 when it first became part of the clerkship.In Figure 2, the same data is represented in a different way; for each of the 12 EBM tasks, the bar represents the student’s level of self-efficacy, or how confident he was that he could perform that task. Beneath each bar is an indication of whether the student got the item correct (indicated by a star), incorrect (indicated by an X), or indicated that they didn’t know and so didn’t answer the question.Figure 3 shows the pie charts that are generated by the justification case. Each colored section represents a theme, and each section’s size depicts the percentage of the student’s clinical decision in that case that was based on that particular theme. Again, the chart on the left is the individual learner’s chart, in the center is the combined data of the current group of participants, and on the right is the chart of all participants since this version of the survey began.

#### Current study

All third year medical students at the University Of Maryland School Of Medicine who completed their pediatrics clerkship between 7/1/09 and 2/11/11 were required to complete the EBM exercise. Students were provided 2 ½ weeks to complete the online exercise located on a secure server at the medical school. After all students in each group completed the exercise, students were provided a personal Learning Profile in pdf format that was to be reviewed prior to attending the debriefing session.

For each student group the clerkship director received a composite report with de-identified results for each student as well as for the group as a whole. Within 3 days of closing the EBM exercise for a cohort, the students attended a one-hour debriefing session during which they had a brief presentation on reflection and its importance in becoming and remaining an excellent physician, and then discussed their own and the group’s results from their profiles. All students were asked to complete an evaluation form following the debriefing session in which they provided feedback on the technical aspects of the exercise as well as the content of the Learning Profile and the utility of the debriefing session. Three of 12 questions included in the debriefing evaluation survey were studied: 1) did the debriefing session help you to better understand your report, why or why not; 2) did the Learning Profile help you understand how you make clinical decisions, why or why not: and 3) based on the information in your report, is there anything you will do differently in the future.

Debriefing data from the student feedback surveys was analyzed using a post-test survey design with a single experimental cohort. Using grounded theory methods [23,24] the debriefing data was analyzed through an iterative process of examining student survey responses. To triangulate data [25] three evaluators (the pediatric clerkship director, the developer of the EBM exercise itself, and the Instructional Technologist who maintains the medical school electronic curriculum) were trained in inductive coding methods and then independently coded survey responses into theme categories, followed by a check-coding and intra-coder procedure where the evaluators discussed theme coding differences. Using refined coding themes and definitions from the first iteration, a second iteration of theme coding was conducted, resulting in 100% agreement. The five themes used to code survey responses are shown in Table 1. The data showing the percentages of survey responses that fall into each of the 5 themes identified through the qualitative methods outlined above are available in the Additional file 1.

**Table 1:** Coding themes and definitions used to analyze student survey responses

**Theme name**	**Definition**	**Example**
Clarification of results	Reported that data graphs, explanation and presentation were clarified in the debriefing session; significance and validity of data presented were put into context	“The graphs and charts were hard to interpret without explanation, but made perfect sense once explained”
Showing group variation	Reported gaining insight into differences among group members; gained awareness of personal ratings	“It helped me compare myself to the rest of the group”
Evaluate myself and decision making	Reported personal insights regarding the use of EBM; visualization, thinking, and clarification about EBM related to decisions	“Gave me a good perspective on constructive ways to evaluate myself and decisions throughout my career”
Understanding EBM exercise purpose	Reported improved understanding of purpose and significance of exercise, alternative ways to learn EBM basics	“Putting the information in a broader context of decision making was helpful”
Other EBM actions	Reported actions that will be pursued by the student that include collaborative decision making, EBM readings, observation of others, gaining confidence in abilities, do nothing and not sure	“make more effort to understand and interpret data,” “ask more questions”

The protocol was deemed exempt by the University of Maryland Institutional Review Board.

## Results

Two hundred and sixty-two third year medical students completed their pediatrics clerkship during the study period. Of those, 224 (86%) completed the EBM exercise. Reasons for not completing the exercise included technical difficulties with the online program and personal attributes leading to lack of timeliness. Two hundred and thirty (88%) of the total number of students attended a debriefing session. One group of 20 students did not have a debriefing session due to closure of the medical school campus for extreme weather. Other students who missed debriefing sessions did so for a variety of personal reasons including illness and competing obligations. One hundred and seventy-one of the students who attended a debriefing session (74%) completed and turned in a survey.

Of the students responding to the debriefing evaluation survey, 95% of the 170 respondents indicated that the debriefing helped them better understand their Learning Profiles, with 45% offering an explanation supporting their response. The vast majority of responses indicated that having the debriefing session clarified the purpose of the EBM exercise and the students’ individual results.

When asked whether the learning profile helped them understand how they made clinical decisions, 68% of the 171 respondents indicated that it had, and of those 40% offered explanations. The majority of explanations stated that the profile allowed them to evaluate themselves and their decisions.

108 (63%) of students demonstrated reflection by providing explanations about what they would do differently based on the EBM exercise debriefing experience. These were split evenly between 1) learning more about EBM and using EBM more in the future, and 2) reflecting more on their own decision making.

Table 2 details student responses to the three questions coded into the five coding themes.

**Table 2:** Descriptive summary of debriefing evaluation survey by coding theme

	**Debriefing helpful? Why/Why Not?**	**Profile help with clinical decisions? Why/Why Not?**	**What will you do differently?**
	N = 77*	N = 68*	N = 108*
Coding theme			
Result clarification # (%)	50 (65%)	22 (32%)	2 (2%)
Group variation # (%)	7 (9%)	5 (7%)	0
Evaluate self & decisions # (%)	8 (10%)	37 (54%)	54 (50%)
Understand EBM purpose # (%)	12 (16%)	2 (3%)	0
Other EBM actions # (%)	0	2 (3%)	52 (48%)

*N = number of Yes or No responses that provided an explanation.

## Discussion

The EBM exercise was originally conceived as a way to study physicians’ attitudes toward evidence based medicine. In its revised form it has served as a reliable way to engender reflection in third year medical students, with two thirds of those completing post-debriefing surveys describing the ways that reflecting on the data provided would lead them to do something differently. Providing a Learning Profile that includes graphic representations of students’ thought processes has led to fascinating discussions that support previous work suggesting that reflection is best fostered in a supportive environment with a facilitator where learners are addressing real situations that potentially create dissonance between internal values and actual performance. This is particularly evident when reviewing student self-efficacy vs. skill graphs (Figure 2).

Although the self-assessment literature suggests that people tend to over-estimate their abilities, self-efficacy is a more targeted measure of “personal judgments of one’s capabilities to organize and execute courses of action to attain designated goals” [26]. Most students in this study were found to underestimate their EBM skills; the reason for this is not clear, and might warrant further study.

The students themselves present some interesting hypotheses when this question is raised in the debriefing sessions, stating that they are more comfortable when they underestimate their abilities and then are found to be better than they thought, rather than overestimating and feeling badly when their skills are less than they had hoped. This supports the notion that individuals may use low expectations as a strategy for lessening anxiety in risky situations [27]. The reflection and discussion around this issue is often lively, leading to a debate whether underestimation is better or worse than overestimation, and how each can be either a strength or a weakness.

The clinical case pie charts led to a different kind of reflection, as there is no “right answer” to how one’s result should look. This was clearly very uncomfortable for many students, who generally have not encountered ambiguity in quantitative exercise results before. Furthermore, showing students several very different pie charts generated by classmates in the same group created an element of surprise and discomfort that also encouraged reflection. A common topic of discussion was how large a contribution to patient care decisions should be made by patient needs and wants, particularly when the scientific evidence is reasonably clear about the most efficacious option. Their reflection often moves to how physicians should interact with patients when they disagree, and how they have seen those situations handled during their clinical rotations. Students become quite engaged in these discussions, and their interest is again reflected in their responses to the post-session survey questions.

Use of graphic representations of students’ individual approaches to the EBM exercise is a hybrid between the direct and indirect approaches to enhancing self-awareness described by Benbassat et al. [28]. He separates direct curricula that include classroom activities where students reflect in a group on their emotional responses to clinical situations from indirect methods where students are asked to reflect on their performance of clinical skills and compare them to the assessments of their instructors or patients. The EBM exercise debriefing has the group discussion component of a “direct” method, but students reflect on visually tangible representations of their performance, which takes away some of the discomfort of discussing emotions with one’s peers.

The EBM exercise itself has some limitations. The self-efficacy and skill section provides only one opportunity for students to complete each EBM task, and it may not be a valid measure of their actual abilities. The justification case produces pie charts of decision making processes that may not be constant in each individual across a range of case types. Additionally, the pie charts themselves are not meant to present a validated representation of data, but rather the relative use of various types of evidence when considering a single case, and are most useful to date in prompting reflection and discussison. With these limitations clearly named for the students at the beginning of the debriefing session, the exercise still presents enough data that students are willing to reflect on what it might mean about their skills.

The survey used to conduct this study also has limitations. First, results from only one medical school may limit the generalizability of the study, and a small number of students (up to 38) who completed the survey had not done the EBM exercise, potentially skewing the results. Those students, however, were able to see results from their classmates and participate in the group discussion, so it is likely that they were still able to reflect, at least in the abstract, on the process of clinical decision making. Also, the use of a survey to determine whether students plan to increase their use of EBM or self-reflection in the future does not prove that they actually do. Ultimately, documenting true behavior change would be a stronger outcome to report.

The grounded theory methodology used to code survey responses adds the risk of data interpretation errors, such as evaluators imposing their own meanings or not considering discrepant data. This study attempted to mitigate these risks using triangulation methods but it is possible that alternative coding categories could reveal discrepant results.

While this exercise clearly prompts reflection in students rotating through pediatrics, it is a very small part of the clerkship curriculum and an even smaller component of the overall medical school experience. In fact, because it is such a small and isolated part of their curriculum at the present time, it may be unlikely to have done more than prompt a one-time reflective process. It is likely that there would be more impact of such an exercise as a part of an overall curricular approach to fostering reflection across the medical school curriculum, with a variety of opportunities to engage in guided reflection.

## Conclusions

In conclusion, introducing the concept of reflective practice via a EBM exercise as part of a third year pediatrics clerkship was successful, and the graphic Learning Profiles were effective instigators of discussion and reflection. Future directions include creating a template and methodology so that a variety of clinical cases could be used in this exercise, broadening its use to disciplines other than pediatrics, and potentially studying whether an individual’s approach to one case predicts his/her approach to other cases. Those who would like to use the current exercise can do so by accessing it online at http://www.docdp.org/ebm/ebm.aspx?id=1&group=1&mode=delivery.

## Abbreviations

EBM: Evidence based medicine.

## Competing interests

The authors declare that they have no competing interests.

## Authors’ contributions

LOL made substantial contributions to the conception and design of this study, acquired and helped analyze the data, and drafted the manuscript. NJR made substantial contributions to the conception and design of the online EBM exercise, analyzed the data, reviewed the manuscript critically, and approved the version being submitted. JR made substantial contributions to the online platform with which the EBM exercise was presented to learners, helped analyze the data, reviewed the manuscript critically, and approved the version being submitted. CC made substantial contributions to developing the EBM exercise and Learning Profile, helped analyze data, reviewed the manuscript critically, and approved the version being submitted. PH made substantial contributions to developing the EBM exercise and Learning Profile, helped analyze data, reviewed the manuscript critically, and approved the version being submitted. All authors read and approved the final manuscript.

## Authors’ information

LOL is Associate Chair for Education and Associate Professor, University of Maryland School of Medicine.

Dr. Robert is Executive Vice President and Chief Product and Marketing Officer, American Nurses Association.

Mr. Raczek is Web Developer, University Of Maryland School Of Medicine.

Dr. Carraccio is Vice President for Competency-based Assessment, American Board of Pediatrics.

Dr. Hicks is Professor of Clinical Pediatrics, Perelman School of Medicine at the University Of Pennsylvania.

## Pre-publication history

The pre-publication history for this paper can be accessed here:

http://www.biomedcentral.com/1472-6920/14/164/prepub

## Supplementary Material

Additional file 1 Responses of Students to Survey Questions by Theme. Click here for file
