# Supplementary material for: An online evidence based medicine exercise prompts reflection in third year medical students
Source: BMC Med Educ. 2014 Aug 9;14:164. doi: 10.1186/1472-6920-14-164 (PMC4132283; doi:10.1186/1472-6920-14-164)
Supplement: Additional file 1 — Responses of Students to Survey Questions by Theme. [file 1472-6920-14-164-S1.docx]

| **Category Definition** | **Was debriefing helpful?** | **Will the profile help with clinical decisions?** | **What will you do differently?** |
| --- | --- | --- | --- |
|  | Q7 | Q9 | Q11 |
| CATEGORY 1-Result Clarification | 50 | 22 | 2 |
| CATEGORY 2-Group Variation | 7 | 5 | 0 |
| CATEGORY 3-Evaluate Self & Decisions | 8 | 37 | 54 |
| CATEGORY 4-Understanding EBM Purpose | 12 | 2 | 0 |
| CATEGORY 5-Other EBM Actions | 0 | 2 | 52 |
| TOTAL ITEMS EXPLAINED | 77 | 68 | 108 |
| PERCENTAGE EXPLAINED | 45.29% | 39.77% | 96.43% |
|  |  |  |  |
| TOTAL ITEMS NOT EXPLAINED | 92 | 99 | 4 |
| PERCENTAGE NOT EXPLAINED | 54.12% | 57.89% | 3.57% |
|  |  |  |  |
| Number YES response | 162 | 116 | NA |
| PERCENTAGE YES | 95.29% | 67.84% | NA |
| Number NO response | 5 | 47 | NA |
| PERCENTAGE NO | 2.94% | 27.49% | NA |
| Number BLANK response | 3 | 8 | 65 |
| PERCENTAGE BLANK | 1.76% | 4.68% |  |
|  |  |  |  |
| OMITTED ITEMS from Themes-not decipherable | 1 | 4 | 4 |
|  |  |  |  |
| NUMBER ITEMS INTER-RATER DISCUSSION | 14 | 32 | 27 |
|  | 8.24% | 18.71% | 24.11% |
|  |  |  |  |
|  | N-170 q7 RESPONDENTS | n=171 Q9 RESPONDENTS | N-112 q11 RESPONDENTS |
